# Editorial: New Developments in Agrobacterium-Mediated Transformation of Tree Fruit Crops

**DOI:** 10.3389/fpls.2019.01253

**Published:** 2019-10-08

**Authors:** Vladimir Orbović

**Affiliations:** Citrus Research and Education Center, University of Florida, Lake Alfred, Florida, United States

**Keywords:** woody fruit crop, *Agrobacterium*-mediated transformation, genetically modified plants, breeding, molecular biology

Because of increased pressure coming from emerging diseases, climate change, growing human population, and decreasing area for agricultural use crops (Gottwald, 2010;Fiore et al., 2018), the need for development of woody fruit crops (WFCs) with improved traits that fit principles of sustainable production is bigger than ever. With conventional breeding techniques being slow as they are, WFC breeders have turned towards molecular techniques for cultivar improvement. One of the most popular techniques of molecular breeding is introduction of genes into the genome of plants with the help of *Agrobacterium tumefaciens*. For the *Agrobacterium*-mediated transformation of WFC species it is necessary to: define methods for high organ regeneration from explants, have working delivery system for genes/sequences of interest, and to be able to do stringent selection of modified shoots. While *Agrobacterium* has proven itself as reliable vector and selection methods have evolved into reasonably good tools, the ability of explants from WFC species to regenerate shoots remains a major impediment to genetic modification (GM). Although genetic transformation of WFCs has been tried for the last 30 years, more than 95% of species that belong to this group are still recalcitrant to transformation. Despite the progress made with citrus, apples, plums, kiwi, pears, cherry, grapes, and blueberry, there are still no reports of production of transgenic plants expressing a gene involved in trait improvement for such important fruit crops like mangoes or peaches.

The mini review within this research topic (RT) (Song et al.) provides a brief historical summary of what was accomplished until now but also describes the directions and challenges to make *Agrobacterium*-mediated transformation of WFCs more successful in the future. The authors of the review list ectopic expression of introduced genes (both trans- and cis-genes), multiple ways of gene silencing within the host, transgrafting, and clustered regularly interspaced short palindromic repeats (CRISPR)-mediated genome editing as a context within which transgenic plants can be used. The work reported in the other papers within this RT follows well the list from the mini review.

By using the thaumatin II gene from the tropical plant *Thaumatococcus daniellii,* Timerbaev et al. wanted to improve the sweetness of apple fruit. Furthermore, the transgenic plants they produced were marker-free due to excision of the part of transfer-DNA (T-DNA) with the *CodA-nptII* gene. This group employed the system that included recombinase R from yeast that is inducible with 5-fluorocytosine (5-FC). Resulting transgenic plants were grafted on the rootstock, which promotes early flowering for quick evaluation of fruit sweetness.

Another paper in this collection reported expression of a foreign sequence into plum trees. In this case, the sequence complimentary to the portion of plum pox virus (PPV) sequence was organized in such a way as to create an intron-containing hairpin structure upon transcription (Sidorova et al.). The presence of intron-hairpin RNA (ihpRNA) in the cells of transgenic plums made them resistant to PPV infection through the process of silencing. Branches from infected trees were grafted onto transgenic plants expressing PPV complimentary sequences. Despite its continuous presence in the grafted branches, PPV never spread in transgenic plum trees within the period of 9 years.

Transgrafting refers to grafting of the non-GM scion on the GM rootstock. These plants exploit the benefit of transgenic technology, although aerial parts that bear fruit are actually not GM. One project using transgrafted plants was described in the paper by Dandekar et al. Rootstock grape plants were modified in two ways: 1) to produce anti-microbial protein and 2) to produce an inhibitor of the breakdown of the cell wall component. In both cases, these proteins were engineered to be secreted out of the cells and ended up in the xylem vessels conducting water and mineral salts up the plant to non-GM scions. Field-grown transgenic grape plants expressing these two genes exhibited 30–95% decrease in mortality after infection with bacteria inducing Pierce’s disease.

The CRIPSR-based methodology was represented with two papers in this RT. In one paper by Charrier et al., the first use of CRISPR-mediated gene editing is reported for pears. These authors also worked with apples and made CRISPR-mediated “edits” in the gene responsible for carotenoid synthesis, which led to pale phenotype. The use of two guide RNAs for each targeted gene increased the possibility of a successful editing event. In the paper by Ren et al., further efforts to improve efficiency of CRISPR-mediated genome editing were described. For two grape cultivars, Chardonnay and 41B, it was shown that the efficiency of CRISPR-mediated gene editing was higher with the high percentage (up to 65%) of GC content in the guide RNAs.

From a quick look at publications in this RT, it is clear that CRISPR-mediated genome editing is quickly overtaking the world of WFC improvement (Charrier et al. and Ren et al.) Besides being a very fashionable thing to do, it is also the technique that raises hopes that “high precision–no insertion” GM will lead to a higher rate of production and acceptance of GMOs by the general public. However, additional arguments for acceptance of “classical” GM plants should be the results presented in other papers from this RT, like long-term protection against pathogen achieved in plums (Sidorova et  al.) or the use of GM rootstocks for transgrafting in grapes (Dandekar et al.).

The work on organogenesis/regeneration, which is the major obstacle to wide application of GM in WFCs, is absent from this RT. Although the research leading to definition of tissue culture methods to achieve regeneration from a single cell is important not only for GM (Kole and Hall, 2008) but also as an alternative way of propagation, almost no efforts are invested to accomplish this. My hope is that this will change in the foreseeable future because there is a lot of work that can be done to assist fruit growers to improve crops that are recalcitrant and those that are amenable with the help of *Agrobacterium*-mediated transformation. Such improvements could make existing elite cultivars of WFCs resistant to diseases, make them more tolerant to abiotic stress, and introduce traits including different fruit color or taste available only in specialty crops.

## Conflict of Interest

The author declare that the research was conducted in the absence of any commercial or financial relationships that could be construed as a potential conflict of interest.
